# Investigation *in vitro* Expression of *CatSper* Sub Fragment followed by Production of Polyclonal Antibody: Potential Candidate for The Next Generation of Non Hormonal Contraceptive

**Published:** 2012-12-12

**Authors:** Mahboobeh Nazari, Manouchehr Mirshahi, Seyed-Javad Mowla, Taravat Bamdad, Sina Sarikhani

**Affiliations:** 1. Department of Biochemistry, Faculty of Biological Sciences, Tarbiat Modares University, Tehran, Iran; 2. Department of Recombinant Technology Research, Nanobiotechnology Research Center, Avicenna Research Institute, Academic center for Education, Culture and Research (ACECR), Tehran, Iran; 3. Department of Genetics, Faculty of Biological Sciences, Tarbiat Modares University, Tehran, Iran; 4. Department of Virology, Faculty of Medical Science, Tarbiat Modares University, Tehran, Iran

**Keywords:** CatSper, Sperm Cation, Male Fertility, Contraception, Antibody

## Abstract

**Objective:**

*CatSper* is a voltage-sensitive calcium channel that is specifically expressed in the testis and it has a significant role in sperm performance. *CatSper* (1-4) ion channel subunit genes, causes sperm cell hyperactivation and male fertility. In this study, we have explored targeting of the extracellular loop as an approach for the generation of antibodies with the potential ability to block the ion channel and applicable method to the next generation of non-hormonal contraceptive.

**Materials and Methods:**

In this experimental study, a small extracellular fragment of *CatSper1* channel was cloned in *pET-32a* and *pEGFP-N1* plasmids. Then, subsequent methods were performed to evaluate production of antibody: 1) *pEGFP-N1/CatSper* was used as a DNA vaccine to immunize Balb/c mice, 2) The purified protein of *pET-32a/CatSper* was used as an antigen in an enzyme-linked immunosorbent assay (ELISA) and western- blot, and 3) The serum of Balb/-c mice was used as an antibody in ELISA and western-blot. The statistical analysis was performed using the Mann Whitney test.

**Results:**

The results showed that vaccination of the experimental group with DNA vaccine caused to produce antibody with (p<0.05) unlike the control group. This antibody extracted from Balb/c serum could recognize the antigen, and it may be used potentially as a male contraception to prevent sperm motility.

**Conclusion:**

*CatSpers* are the promising targets to develop male contraceptive because they are designed highly specific for sperm; although, no antagonists of these channels have been reported in the literature to date. As results showed, this antibody can be used in male for blocking *CatSper* channel and it has the potential ability to use as a contraceptive.

## Introduction

Study of ion channels in biology has a special significant effect. Ability to regulate ion channels control the membrane potential and intracellular concentrations of ions like calcium which leads them to play an important role in cellular processes that control the excitability of the motor, such as stimulus-secretion coupling, regulation of cell volume and electrolyte transport epithelium. The importance of calcium ions in many functions, such as movement of the sperm and acrosome reaction are well marked (1-4). Despite the fact that several voltage-gated channels localized in the sperm have been cloned and expressed, the molecular nature of ion channels and ion transport mechanisms involved has only recently begun to emerge (5).

The search for Ca^2+^ channels residing in sperm has showed a path to the cloning and identification of a novel gene, called *CatSper*. *CatSper* codes a Ca^2+^ channel specifically expressed in the testis (6). It has an important role in sperm motility, sperm penetration into oocyte, and finally in male fertility (6). It has shown that the expression of *CatSper* is not only developmentally regulated, but also significantly reduced in subfertile men having impaired sperm motility (6). *CatSper1* and *CatSper2* are voltage-gated ion channels with putative six-transmembrane settled on the sperm flagellum (6-10). Moreover, the different studies on gene targeting have revealed that both mentioned-genes are required for cAMP-induced Ca^2+^ current which is essential for normal sperm motility and male fertility (7). Ren et al., 2001, has described a new method to block sperm motility and sperm hyperactivation based on the function of a group of the four novel proteins located on transmembrane of the calcium channels, namely *CatSper* (7). Furthermore, the other studies on primates and rodents have indicated the important physiological roles channel-like protein and the possible presence of *CatSper1* in sperm competition (11, 12).

While the world population continues to increase, there is an urgent need to control the growth rate. So, many efforts have been formed to develop safe, effective and reversible male contraceptive. In the last decades, various approaches have been used to develop hormonal and non-hormonal contraceptive (13-15). Several attractive methods for non-hormonal male contraceptive are as follows: I. Reversible inhibition of sperm under guidance (RISUG) which partially blocks vas deferens (16), II. Indenopyridines which affects the germ-cell adhesion (17), III. Gossypol which affects the leydig-cell steroidogenesis (18), IV. Vaccines such as Eppin, which functions in semen liquefaction (19), and V. Calcium channel blockers which affects sperm membrane cholesterol (20). Among these, the ion channel has become a very interesting subject for the researchers because it is considered to be as a target to design anti-fertility drugs to prevent pregnancy. According to the previous researches, some channels are exclusively expressed in sperm, thus their selective knock-down leads to infertility with no adverb effects. Recently, inhibition of sperm motility as a favorable target in development of male contraceptive has been investigated by the different pharmaceutical companies (21). The main mechanism of male contraceptive is that the sperm becomes "hyperactive" prior to fertilization which means the sperm flagella motility exhibits an asymmetrical whip-like beating pattern. Hyperactivated motility enables sperm to penetrate the ovum’s cumulus oophorus and zona pellucid (22). Inhibition of sperm motility, hyperactivation, or both is an efficient method for male contraception. An appropriate drug targeting the motility or hyperactivation of sperm is expected to separate into seminal fluid and to ejaculate with the sperm; although, it would not necessarily pass the "blood-testes" barrier. Furthermore, a sperm motility inhibitor may show a very rapid onset of action and acts as a contraceptive immediately before intercourse.

To the best of our knowledge, no contraceptive based on *CatSper* blocking has ever been reported. In the present study, we have cloned a small segment of *CatSper* gene that codes for extracellular domain of a protein with no significant similarity with other known Ca^2+^ channels. This segment was cloned in two different expression vector, one of them is a eukaryotic vector pEGFP-N1 containing the human cytomegalovirus (CMV) promoter and the other one is green fluorescent protein (GFP) coding sequences as a reporter gene which was used to immunize animal models, a DNA vaccine. The latter one, prokaryotic expression vector *pET-32a*, was used for expression of this segment in E. coli BL_21_. Then, the production of antibody was evaluated by immune-blotting.

## Materials and Methods

For this experimental study, we used the following reagents and kits: isopropyl-β-D-thiogalactopyranoside (IPTG), kanamycin, Ampicilin, T4 DNA ligase, (*BamHI* and *HindIII*) restriction enzyme (Fermentas, Canada), plasmid extraction kit, gel purification kit, Ni-NTA spin kit (QIAGEN Inc, Venlo, Netherlands.), *pET-32a* vector (Novagen, Darmstadt, Germany), Dulbecco’s modified eagle’s medium (DMEM) high glucose (BioSera, Gentaur, Austria), Bl21 (DE3) and DH5-α (Novagen, Darmstadt, Germany), Chinese Hamster Ovary cells (CHO), (Life Technology, California), Fetal Bovine Serum (FBS), (GIBCO, Grand Island, NY), LipofectamineTM 2000, (Invitrogen, Grand Island, USA), phenylmethylsulfonyl fluoride (PMSF), (Sigma-Aldrich, Canada), Bovine Serum Albumin (BSA), (Sigma-Aldrich, Canada), Phosphate Buffer Saline (PBS), (Takara, France), goat anti mouse IgG conjugated to horseradish peroxidase (Invitrogen, Grand Island,USA), diaminobenzidine (DAB), (Ameresco, USA) and *pEGFP-N1* plasmid (Clontech, USA). All data presented in this manuscript were repeated at least three times. Also, they are considered to be the typical experimental data.

### Designing synthetic oligos (the minigene)

Using the gene bank database (Acc. No NP_647462.1), we clarified the sequence (between S1 and S2) in the Ca^2+^ channel is FTELEIRGEWTF. Since the minigene of interest was too short to be amplified by the polymerase chain reaction (PCR), two sets of oligo-nucleotides were designed to be inserted into two plasmids. Regarding the multiple cloning sites on *pET-32a*, two sets of oligo P1 and P2 without ATG codon were designed containing *BamHI* and *HindIII* with overhangs at the 5´and 3´ ends, respectively. For *pEGFP-N1* plasmid, another two sets of oligos, P3 and P4, with the kozak site (ACCATGG) at the 5´ ends was designed to enhance eukaryotic translation. These oligos contain *HindIII* and *BamHI* with overhangs at the 5´ and 3´ ends, respectively. The synthetic fragments were not phosphorylated at the 5´ ends to eliminate the possibility of tandem formation. The MWG-Biotech Co. (Germany) provided these synthetic oligos with 45 bp for prokaryotic and 53 bp for eukaryotic expression vectors (Table 1).

### Plasmid ligation strategy

After annealing oligos with gradient thermal programs, the cut site for *BamHI* and *HindIII* were constructed. Afterwards, these oligos were inserted into the *BamHI*-*HindIII* restriction sites of the digested *pET-32a* (+) and *pEGFP-N1* to achieve highly-expression plasmid vectors, then the ligated mixtures were transformed into the competent cells of *Escherichia coli* DH5-α by an electroporation.

**Table 1:** The sequences of oligo were used for cloning in prokaryotic and eukaryotic plasmids


Synthetic oligos for prokaryotic vector	Sequence

P1 (forward) containing the BamHI	5' GATCC TTC ACT GAG CTA GAG ATC CGA GGT GAA TGGTACTTCTAG A 3'
P2 (reverse) containing HindIII	5' AGCTTCTAGAAGTA CCA TTC ACC TCG GAT CTC TAG CTCAGTGAA G 3'
After annealing P1 and P2 oligo, the minigene is:	5´ GATCC TTC ACT GAG CTA GAG ATC CGA GGT GAA TGGTACTTCTAG A 3´
	3´ G AAG TGA CTC GAT CTC TAG GCT CCA CTT ACC ATGAAGATCTTCGA 5´
Synthetic oligos for eukaryotic vector	Sequence
P3 (forward) containing HindIII	5' AGCTTACCATGGCA TTC ACT GAG CTA GAG ATC CGA GGT GAA TGG TACTTCGCG 3'
P4 (reverse) containing BamHI	5' GATCCGCGAAGTA CCA TTC ACC TCG GAT CTC TAG CTC AGTGAA TGG CATGGTA 3'
After annealing P3 and P4 oligo, the minigene is:	5´ AGCTTACCATG GCA TTC ACT GAG CTA GAG ATC CGA GGTGAA TGG TACTTCGCG 3´
	3´ ATGGTAC CGT AAG TGA CTC GAT CTC TAG GCT CCA CTTACC ATGAAGCGCCTAG 5´


The sequence of restriction enzyme is underlined, and kozak sequence introduced in eukaryotic plasmid is italicized.

### Sequencing

*pET-32a* (+) vectors containing synthetic oligo were sequenced using an automatic sequencer (MWG) by the T7 promoter universal primer; whereas,* pEGFP-N1* vector containing synthetic oligo was sequenced using an automatic sequencer (MWG) by *EGFP-N* sequencing primer.

### Obtaining the cell containing the GFP plasmid

The Chinese hamster ovary cells (CHO) was grown in DMEM high glucose supplemented with 10% fetal bovine serum (FBS). Transfection procedure was performed by LipofectamineTM 2000 as described by the manufacture’s manual (Invitrogen life technologies, USA). The transfected cells were incubated at 37 ºC with 5% CO_2_ for 24 and 48 hours, respectively. Last, the transfected cells were checked with an inverted fluorescence microscope for the cells containing the GFP plasmid.

### Animal vaccination

For DNA vaccination, Balb/c male mice (aged=6-8 weeks old; n=21 and 400 g weight) were obtained from the Pasteur Institute (Karaj, Iran). Balb /c mice were maintained under the standard conditions with free access to water and rodent laboratory food. Then, they were divided into the three groups: 1. The experimental group including eight mice were vaccinated with 50 µg of the constructed DNA vaccine (*pEGFP-N1/CatSper*) diluted in phosphate buffer saline (PBS). The control group including 5 mice were injected with PBS 3. The placebo group eight mice was vaccinated with 50 µg empty vector *(pEGFP-N1)*. The vaccination was applied subcutaneously three times with two-week intervals. Two weeks after the final injection, the serum was obtained from the tail artery of mice. Moreover, the pre-immune serum sample was used as the negative control. Since Balb/c mice are inbred and have the same genetic background, a similar immune response was expected to occur. In this study, the variations of the results are minimized due to use a set of at least five mice in each group. All the plasmids were used for mice immunization were purified with an endotoxin-free plasmid mega kit (Qiagen, Venlo, Netherlands).

### Using pET-32a for expression and purification of the recombinant CatSper peptide

In order to express and to purify the recombinant *CatSper* peptide (between S1 and S2), a segment was cloned in *pET-32a*. The thioredoxin/*CatSper* fusion protein was expressed in *Escherichia coli* BL21. Five mL of terrific broth (TB) medium containing 100 µg ml^-1^ Ampicilin with a fresh bacterial colony harboring the expression plasmid was incubated at 37℃ during overnight. Then, 500 µL of the overnight cultures with 200 mL of the medium was incubated at 37℃ with vigorous shaking until the OD_600_ reached 0.9. Subsequently, IPTG was added to the solution to a final concentration of 1mM followed by incubating, the mixture at 25℃ for 5 hours with vigorous shaking. In order to find the appropriate condition of the expression, IPTG and lactose were added to the mixture in the serial dilution steps, so the minimum and maximum concentration of IPTG (with or without lactose) were 0.005 mM and 1 mM, respectively. Then, the mixture was incubated at the different temperature time series (18℃- 37℃ in 5 hours- 20 hours). Consequently, the best condition for the protein expression was the above-mentioned procedure. The cells were harvested by centrifugation at 5000 g in 15 minutes. The cell pellet was resuspended in a lysis buffer (50 mM Tris-HCl, 300 mM NaCl, 10 mM imidazole, and 1 mM phenylmethylsulfonyl fluoride (PMSF) (freshly added at pH=7.8). Purification of His6-tagged fusion protein was performed with the Ni-NTA spin column as described by the manufacturer’s manual (Qiagen, Venlo, Netherlands).

### Electrophoresis and western blotting

Purified *Trx/CatSper* was separated on 12% sodium dodecyl sulfate-polyacrylamide gel electrophoresis (SDS-PAGE) by the explained method of Laemmli (23). The proteins were then transferred to nitrocellulose membrane using the method described by Towbin et al. (24). Then, the membrane was blocked with 1% bovine serum albumin (BSA) followed by incubating with the serum of animals at 4℃ during overnight. After three times of washing with PBS, anti mouse IgG conjugated to horseradish peroxidase (1:1000) was added for 1 hour our at room temperature, finally it was incubated with 0.5 mg ml^-1^ of diaminobenzidine (DAB, USA Ameresco) and 0.05% hydrogen peroxide for 10 minutes.

### Enzyme-linked immunosorbent assay for CatSper fragment

The sera from immunized mice were evaluated by the enzyme-linked immunosorbent assay (ELISA) (25-26). So, they were placed in a 96-well polystyrene micro-plate (Immunlon, Dyna tech, India) coated with recombinant *Trx/CatSper* (5 µg/ml) in 50 mM PBS buffer (pH=7.4) and kept at 4℃ during overnight. Next day, after 2 hours of blocking with 10% (v/v) BSA at 37℃, the plates were drained and washed three times with 0.1 mol L-1 PBS containing 0.05% (v/v) Tween 20 (PBST) for 10 minutes. A dilution series of each serum sample was applied to each well and incubated for 1 hour at 37℃. They were washed and incubated with alkaline phosphatase conjugated goat anti-mouse IgG (diluted 1:1000 in PBS) for 2 hours at 37℃, then washed five times as described-above and the bound of phosphatase activity was measured with diaminobenzidine (DAB, Ameresco, Canada). Finally, the plates were incubated at room temperature for 10 minutes and the OD was recorded in an Anthos2020 ELISA-reader apparatus at 450 nm.

### Statistical analysis

Data were expressed as mean ± standard error. Statistical analysis was performed using Mann Whitney test, and the significant level was defined as p<0.05. The data were analyzed by GraphPad Prism 5 statistical software.

## Results

### Cloning the minigene of interest

**Fig 1:**
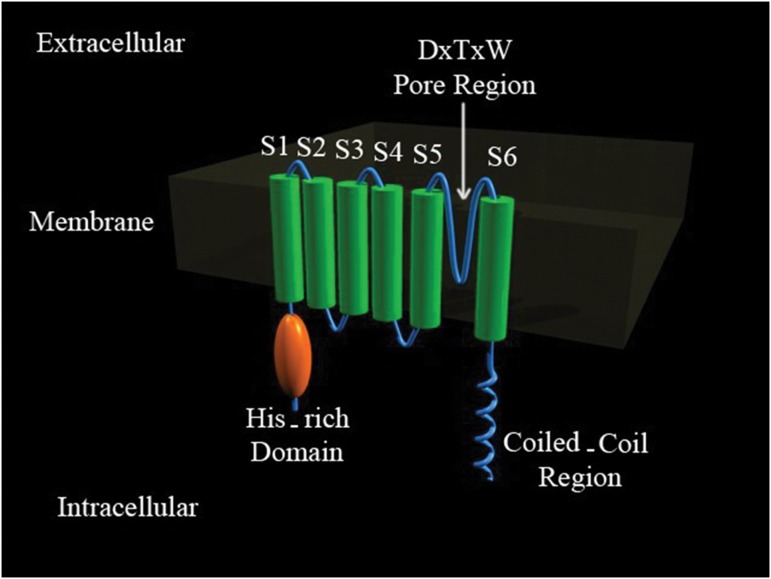
Topology diagram of *CatSper* based on the study of Ren et al. (7, 8). A unique Ca^2+^ channel is expressed exclusively in the testis. The arrow shows the situation of small segment, which was designed and cloned in this study (this figure was developed by applying 3D computer graphics software (maya)).

**Fig 2:**
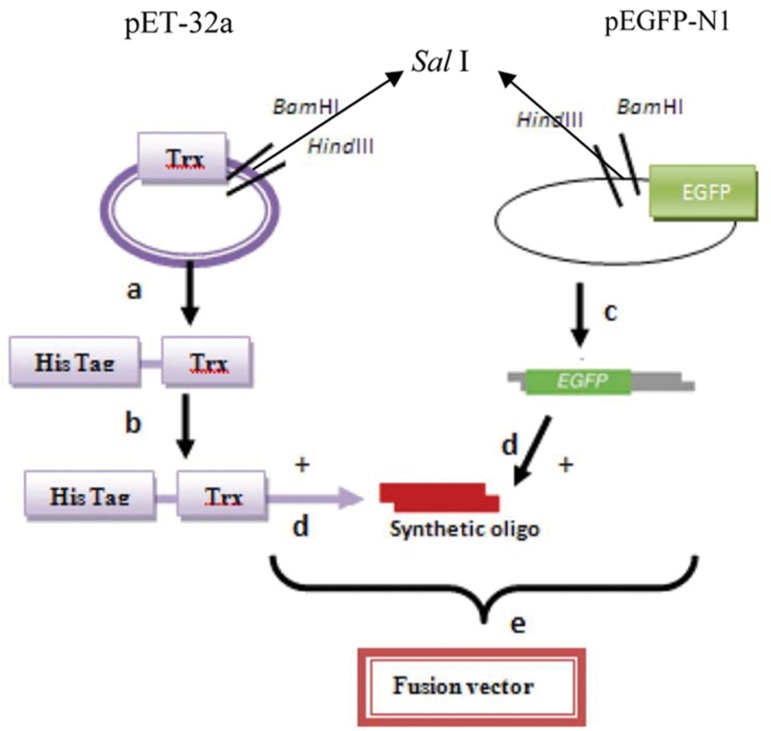
Schematic diagrams of construction the fusion vectors. (a) Digestion pET-32a by BamHI and HindIII. (b) Ligation of pET-32a vector with insertion of *CatSper* fragment (c) Digestion* pEGFP-N1* by BamHI and HindIII. (d) Ligation of *pEGFP-N1* vector with synthetic oligo. (e) Construction of fusion vector.

**Fig 3:**
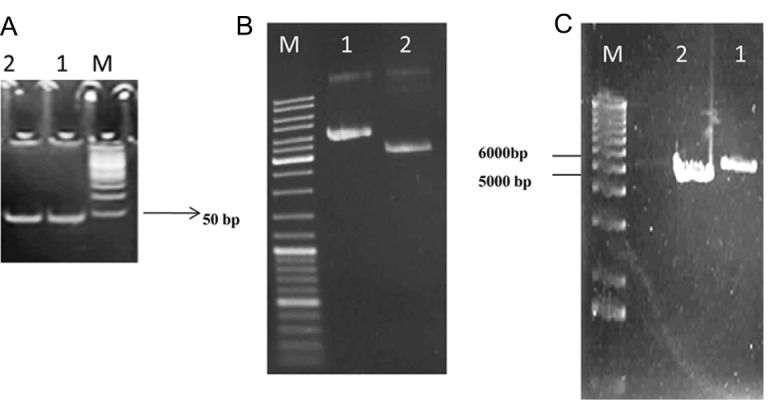
The synthetic of oligonucleotide gene resolved on 12% poly acryl amide gel electrophoresis (Lane 1: synthetic oligo of 45 bp and lane 2: synthetic oligo of 53 bp) (A). Plasmid mini preparation, double digestion with BamHI and HindIII (Lane 1: pET-32a of 5900 bp and lane 2: pEGFP-N1 of 4700 bp) and mono digestion with SalI to confirm cloning (B, C).

As the Topology diagram of *CatSper* shown in the figure 1, the construction process was completed. The figure 2 demonstrates the schematic map of the cloning process. pET-32a and* pEGFP-N1* were digested by *BamHI* and *HindIII* (Fig 3B), then 45 bp and 53bp fragments of mini-gene (Fig 3A) were directly cloned into these vectors, respectively. In the successfully constructed vectors of *pET-32a/CatSper* and *pEGFP-N1/CatSper*, the *BamHI* and *HindIII* sites were impossible to get more cuttings. So, other restriction enzyme, SalI, was selected to demonstrate the recombinant plasmids containing the minigene of interest. Restriction enzyme digestion products were analyzed on an agarose gel (Fig 3C). The positive clones were sequenced followed by the confirmation of the accuracy.

### Expression of mini gene in mammalian cell

**Fig 4:**
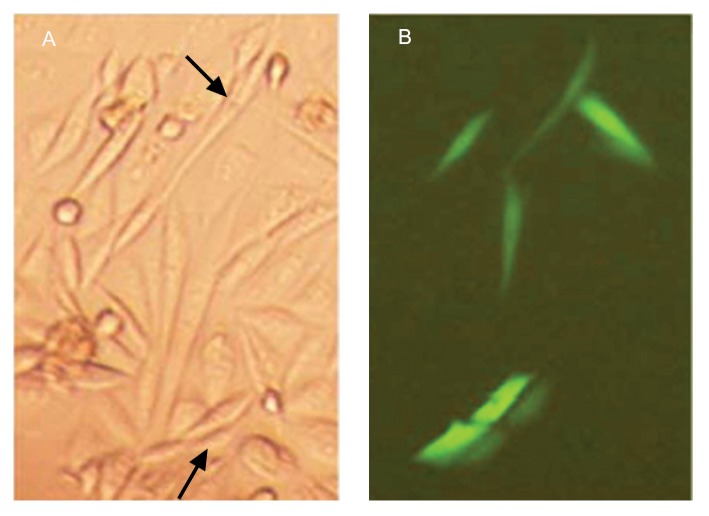
Showing the EGFP expressed from *pEGFP-N1/CatSper* in CHO cells (×40). The cells (1.5×105) in a 6-well tray were transiently transfected with 5 µg of *pEGFP-N1/CatSper*. The cells were observed under a fluorescent microscope (Olympus, CK-2, Tokyo, Japan) without fluorescent excitation in A and with blue filter in B after 48 hours (Images were taken with a ×40 objective lens).

To understand whether the minigene is expressed in mammalian cells, CHO cell line was transfected with *pEGFP-N1+CatSper*, and then the cells were checked for GFP expression after 24 hours (Fig 4B). The multiple cloning sites (MCS) of *pEGFP-N1* are located at the promoter of CMV (*PCMV IE*) and the *EGFP* coding sequences. Since the minigene of interest was cloned into the MCS of *pEGFP-N1* plasmid which is in the same reading frame as *EGFP* and there are no intervening stop codons, it was expressed as fusions to the N-terminus of *EGFP*. The process of the *EGFP* mRNA 3' end are contorted by *SV40* polyadenylation signals located downstream of the *EGFP* gene. An ideal vector has to contain an *SV40* in order to replicate in mammalian cells expressing the *SV40* T antigen.

### Expression and purification of the minigene

**Fig 5:**
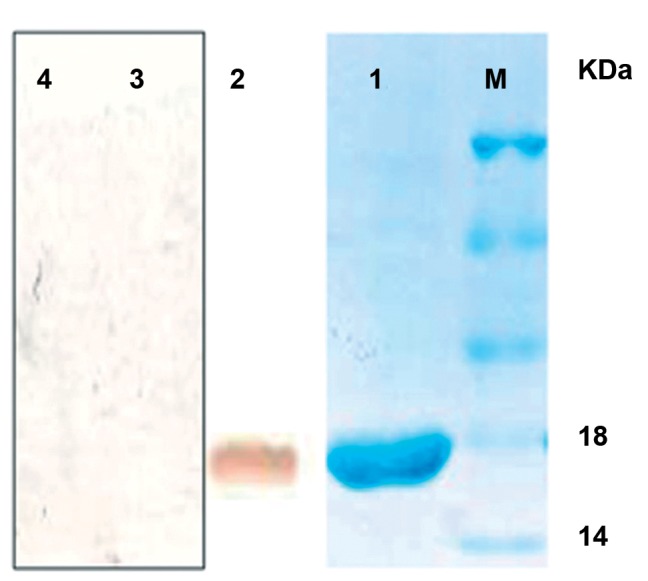
SDS-PAGE of purified Thioredoxin/CatSper and His6 tag from pET-32a with Ni-NTA Sepharose (lane 1). Western blotting of purified synthetic oligo with the serum of the experimental group as the positive control (lane 2), western blotting of purified synthetic oligo with the serum of the control group as the negative control (lane 3), and western blotting of purified Thioredoxin and His6 tags without synthetic oligo with the serum of the placebo group as the second negative control (lane 4) (Marker size 45, 35, 25, 18, and 14 KD).

In order to purify and characterize the minigene, *pET-32a/CatSper* was expressed in the BL21 (DE3) strain. The purification of His-tag fusion protein was also performed by affinity (Ni-NTA-Sepharose) chromatography. *Trx/CatSper* fusion protein was used as antigen in Elisa and western-blot analysis. The recombinant protein was purified and analysed by SDS-PAGE in which fusion protein was present as a band of 18 kDa (Fig 5).

### Immunoblotting

To further characterize antibody production against the interested minigene, the immunoblotting test was done involving mouse IgG conjugated to horseradish peroxidase. After accomplishing the visualization steps, as we expected, there was no bond detected on the nitrocellulose paper for the negative controls (Fig 5). However, the results showed a clear bond while visualizing the resultant fusion protein of the mouse IgG conjugated directly to HRP (Fig 5), which pointed to the successful transfer of immunoglobulins to nitrocellulose paper. The 18 kDa band detected in sera from infected mice provided the antigenicity of *pEGFP-N1+CatSper*.

### Humoral response

**Fig 6:**
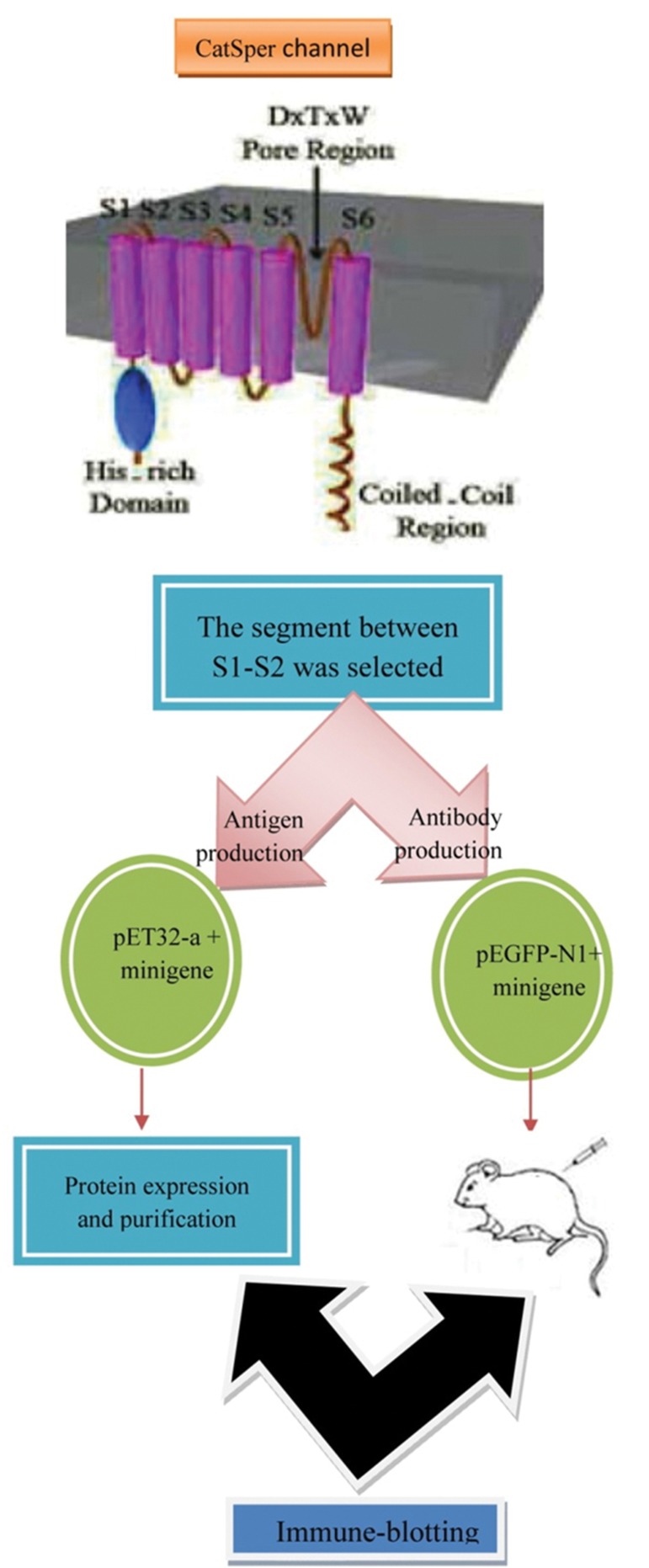
A graphical illustration of the production of antibody against CatSper1 fragment in this study.

The sera from immunized mice were collected and analyzed by ELISA for the response of the specific antibody. Intradermal immunization of *pEGFP-N1/CatSper* in the experimental group resulted in the development of high titers of *CatSper* fragment specific antibody (Fig 6) by applying ELISA with the recombinant Trx/Cat- Sper protein.

**Fig 7:**
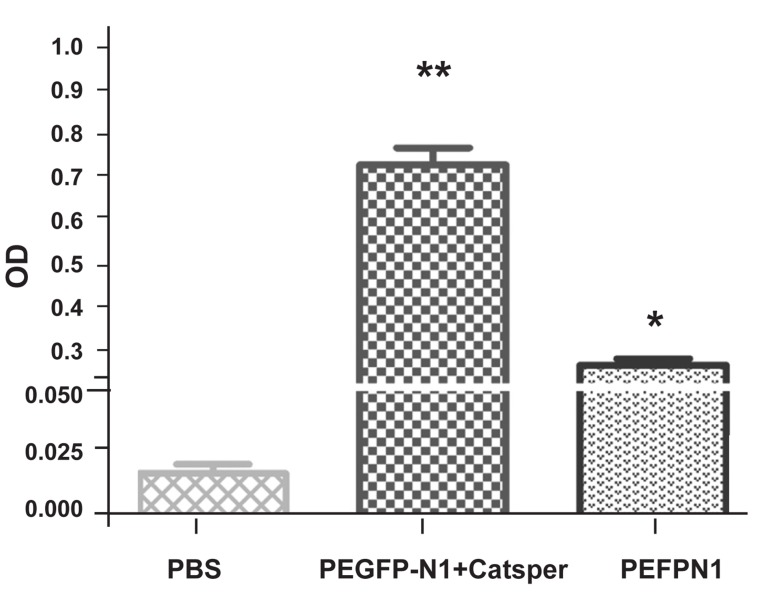
The serum of the antigen-specific total IgG titer following intradermal administration of pEGPN1, *pEGFP/N1/ CatSper* and PBS. Each group is comprised of 5-8 mice. The results are expressed as mean ± SD.

The IgG antibody titer was higher in the sera of mice inoculated with *pEGFP-N1/CatSper* (experimental group) than in the sera of mice immunized with the naked* pEGFP-N1* (control group) or PBS (placebo group). The statistical analysis has revealed the values of, p<0.05, and p<0.008 presented the significant differences between the three groups, experimental and control groups, and control and placebo groups, respectively (Fig 7).

## Discussion

There are several ion channels in a sperm, but most of the recent studied were about the channels enabling the sperm to dissolve the thick outer coating of the egg (27). The study of Clapham et al. has explained that a novel calcium-selective ion channel, named *CatSper*, plays an important role in sperm mobility (28). To analyzing tissue for the distribution and localization of *CatSpers*, many laboratories have applied the multi-tissue northern blot analysis to investigate the gene expression of each member of the *CatSper* family. They have found that *CatSper* 1, 2, 3 and 4 mRNA were expressed exclusively in human and mouse testis (27-29) Other investigations used the antibody staining method to localize the *CatSper* 1-4 protein have proven that *CatSper* 1-4 proteins,which is recognized only in testis and their localization, are in the principal piece of the flagella within spermatozoa (7, 27-29).

There are several promising non-hormonal contraceptive. One of them is sperm-specific Na^+^/H^+^ exchanger (sNHE) which is a trans membrane protein localizing the principal piece of sperm flagella (30). sNHE playa a vital role in osmoregulation, pH control, cell energitics, and sperm function (31). The other one is *CatSper1*, which represents a unique class of putative ion channel-like protein with six transmembrane segments (7). As it is a characteristic for voltage-gated channels, the fourth transmembrane segment of *CatSper1* contains positively charged amino acids interspersed between every three amino acids. Depolarization can evoke Ca^2+^ increase in *CatSper1-/-* sperms, but not in *CatSper1+/+* sperms (10).

In this study, the antigenicity of peptides fragment from a *CatSper* protein were investigated with bioinformatics servers, then based on the solvent accessible regions including both hydrophobic and hydrophilic residues, the solvent accessible segment between S1-S2 with no homology with other Ca^2+^ dependent channel was selected. After cloning and expression of this fragment into plasmids, one of them was used as DNA vaccine (*pEGFP-N1/CatSper*) to immunize the Balb-c. Also, the purified protein of the other one (*pET-32a/CatSper*) was used as an antigen in immunoblotting. The obtained results have shown that this antibody extracted from Balb-c serum could recognize the antigen and it may be used potentially for contraception as it can prevent sperm motility. These results are consistent with the study by liu et al.that they immunized female mice with a DNA vaccine of a testis- specific sodium-hydrogen exchanger channel. Their data showed that IgA in the vaginal fluid and IgG in the serum obtained from the immunized mice were capable of binding to the recombinant protein in HeLa or 3T3 cells. On the other hand, the ELISA assay showed that the DNA vaccine produced in the mice was capable of inducing a high titer of the antibodies, which may be as a result of both humoral and mucosal immune responses (32). The results of this study showed a correlation with the report of liu et al. In fact, the production of antibody against voltage-dependent channels interferes with the sperm motility. It might be a new avenue to explore for a new generation of contraceptive drugs in the future. However, further research with a larger-controlled trial is required before recommendation for a broad clinical application (32).

## Conclusion

The cAMP: PKA signal transduction axis is central to the regulation of ion flux across the sperm plasma membrane and the regulation of hyperactivated movement. Within this regulatory nexus, there are many opportunities for contraceptive intervention. Most of the key players in this process, *CatSper* 1–4, ATP1A4, sNHE, PKAs and sAC, are highly restricted to the male germ line and generate an infertility phenotype when the corresponding gene is subjected to functional deletion. In this study, we conclude that by blocking the *CatSper* channel from action, the sperm will no longer be able to fertilize the egg. Also, upon further study and evaluation, the DNA vaccine may be capable of inhibiting sperm motility and possibly use as contraceptives in men or women.
